# Efficient In‐Cloud Removal of Aerosols by Deep Convection

**DOI:** 10.1029/2018GL080544

**Published:** 2019-01-23

**Authors:** Pengfei Yu, Karl D. Froyd, Robert W. Portmann, Owen B. Toon, Saulo R. Freitas, Charles G. Bardeen, Charles Brock, Tianyi Fan, Ru‐Shan Gao, Joseph M. Katich, Agnieszka Kupc, Shang Liu, Christopher Maloney, Daniel M. Murphy, Karen H. Rosenlof, Gregory Schill, Joshua P. Schwarz, Christina Williamson

**Affiliations:** ^1^ Cooperative Institute for Research in Environmental Sciences University of Colorado Boulder Boulder CO USA; ^2^ Earth System Research Laboratory National Oceanic and Atmospheric Administration Boulder CO USA; ^3^ Institute for Environment and Climate Research, Jinan University Guangzhou China; ^4^ Department of Atmospheric and Oceanic Sciences University of Colorado Boulder Boulder CO USA; ^5^ Laboratory for Atmospheric and Space Physics University of Colorado Boulder Boulder CO USA; ^6^ Goddard Earth Sciences Technology and Research Universities Space Research Association Columbia MD USA; ^7^ Atmospheric Chemistry Division National Center for Atmospheric Research Boulder CO USA; ^8^ College of Global Change and Earth System Science Beijing Normal University Beijing China; ^9^ Now at the Faculty of Physics University of Vienna Vienna Austria; ^10^ School of Earth and Space Sciences University of Science and Technology of China Hefei China

**Keywords:** deep convection, aerosol, convective transport, secondary activation

## Abstract

Convective systems dominate the vertical transport of aerosols and trace gases. The most recent in situ aerosol measurements presented here show that the concentrations of primary aerosols including sea salt and black carbon drop by factors of 10 to 10,000 from the surface to the upper troposphere. In this study we show that the default convective transport scheme in the National Science Foundation/Department of Energy Community Earth System Model results in a high bias of 10–1,000 times the measured aerosol mass for black carbon and sea salt in the middle and upper troposphere. A modified transport scheme, which considers aerosol activation from entrained air above the cloud base and aerosol‐cloud interaction associated with convection, dramatically improves model agreement with in situ measurements suggesting that deep convection can efficiently remove primary aerosols. We suggest that models that fail to consider secondary activation may overestimate black carbon's radiative forcing by a factor of 2.

## Key Points

- Deep convection efficiently removes particles at and above the cloud base
- The default convective scheme in climate model overestimates middle and upper tropospheric aerosol (sea salt and black carbon) by a factor of 10–1,000
- The default convective transport scheme overestimates black carbon's radiative forcing by a factor of 2

## 1 Introduction

Deep convection typically dominates the vertical transport of atmospheric chemical tracers including aerosols (Ervens, 2015). Wet scavenging of aerosols happens both below cloud by Brownian diffusion and impaction and in cloud by nucleation scavenging (activation into cloud droplets). In most chemistry‐climate models (CCMs), activation of aerosols larger than ~50 nm is normally assumed to occur only at the cloud base during primary activation (e.g., Abdul‐Razzak & Ghan, 2002). However, a recent study (Fan et al., 2018) found that even ultrafine particles (less than 50 nm in diameter) are activated above the cloud base in deep convective clouds observed over the Amazon rainforest. Modeling studies (Berg et al., 2015; Ghan et al., 2011; Wang et al., 2013; Yang et al., 2015) show that aerosol nucleation scavenging not just at the cloud base but also above the cloud base is needed to match observed aerosol vertical distributions, but in many cases, such secondary nucleation is ignored in global‐scale CCMs and in regional models.

Convective transport schemes vary in different climate and regional models. In the National Science Foundation/Department of Energy Community Earth System Model (CESM), deep convection is parameterized following Zhang and McFarlane (1995) and shallow convection is parameterized based on Park and Bretherton (2009). In general, the default parameterization scheme for deep convection assumes a stationary state of an ensemble of convective “plumes” among updrafts, downdrafts, entrainment, and detrainment. The shallow convection in CESM is represented by a single updraft plume. While the transport of water, temperature, and momentum by deep convection is widely discussed and validated in previous studies (Zhang & McFarlane, 1995), the transport of tracers by convection is not as well constrained, especially in CCMs. However, in‐cloud removal associated with deep convection is one of the most sensitive processes determining the aerosol mass budget and the aerosol vertical distribution (Grell & Freitas, 2014).

A number of in situ measurements of the aerosol vertical distribution in the upper troposphere and lower stratosphere have been made in recent years. For this study we include the Atmospheric Tomography Mission (ATom) from 2016 to 2017 (Wofsy et al., 2018), the HIAPER Pole‐to‐Pole Observations (HIPPO) campaign from 2009 to 2011 (Wofsy, 2011), and three balloon‐borne particle measurements in 2015 (Gao et al., 2016). Those observations, combined with modeling studies (Koch et al., 2009; Schwarz et al., 2010, 2013; Yu et al., 2017), reveal that global models including the CESM transport too much aerosol from the surface to the upper troposphere and overestimate middle and upper tropospheric mass concentration by factors of up to 1,000. The large discrepancy for black carbon (BC) in the middle and upper troposphere between multiple models and observations (Schwarz et al., 2010, 2013) suggests that wet scavenging is underestimated globally and the large discrepancy among various models (Koch et al., 2009) likely comes from the biases in various convective transport schemes used in different models. Similarly, Yang et al. (2015) show that a regional chemistry transport model (Weather Research and Forecasting‐Chemistry; Grell et al., 2005) underestimates aerosol scavenging (e.g., organics and sulfate) by 30–40% within a supercell storm, while an updated scheme considering secondary aerosol activation from entrained air above the cloud base (similar to the one developed by Berg et al., 2015) improved the model performance, reducing underestimates of scavenging to less than 10%.

There are numerous modeling assumptions in CESM that may lead to errors. The default parameterization scheme in CESM treats wet deposition of aerosols associated with convective clouds separately from the convective transport (Wang et al., 2013). In addition, the wet removal is treated in the large scale (grid‐cell mean), rather than on the convective scale (subgrid) as in Weather Research and Forecasting‐Chemistry model (Grell & Freitas, 2014). Finally, no secondary activation of aerosol from entrained air above the cloud base is simulated.

Here we introduce and validate a modified convective transport parameterization scheme that considers aerosol‐cloud interactions and activation from entrained air above the cloud base within the convective scales. The new parameterization scheme is evaluated using various state‐of‐the‐art in situ measurements of aerosol vertical distributions. The different impacts of the modified scheme on modeled primary and secondary aerosols are presented in section 5.

## 2 Methods

This study uses a sectional aerosol microphysics model, the Community Aerosol and Radiation Model for Atmospheres (CARMA), coupled with the CESM to investigate convective transport of aerosols and trace gases (Toon et al., 1988; Yu, Toon, Bardeen, et al., 2015). CESM/CARMA includes primary particles (BC, mineral dust, sea salt, and primary organics) and secondary particles (sulfate and organics). The model is run at a horizontal resolution of 1.9° × 2.5°, with a time step of 30 min and with 35 vertical pressure levels between the surface and 200 hPa and 21 vertical pressure levels from 200 to 2 hPa. Simulations extend from 2016 to 2017, and model results are compared with multiple observational data sets. Simulations are nudged to Modern‐Era Retrospective analysis for Research and Applications meteorology (Rienecker et al., 2011).

CARMA includes two groups of aerosols. The first group consists of pure sulfate particles whose size distribution is resolved with 20 size bins having diameters ranging from 0.4 nm to 2.6 μm. These particles form through nucleation and condensation of water and sulfuric acid vapor (Zhao & Turco, 1995). The second group consists of internally mixed aerosols whose size distribution is resolved with 20 size bins having diameters ranging from 100 nm to 17 μm. These mixed aerosols are composed of particles containing organics, BC, sea salt, dust, and condensed sulfate. The optical properties of the aerosols in CESM/CARMA are estimated using a core‐shell Mie scattering code based on inputs of particle size, relative humidity, and composition (Yu, Toon, Bardeen, et al., 2015; Yu et al., 2016). Emissions of BC and primary organic aerosol used in the simulations are from the Global Fire Emission Database version 3 for biomass burning sources (van der Werf et al., 2010) and Greenhouse gas‐Air pollution Interactions and Synergies model for anthropogenic sources (Amann et al., 2011).

Most users of CESM employ a modal aerosol module (MAM) to study aerosols (Liu et al., 2012). We also investigate the role of convective removal in the three‐mode MAM, which includes Aiken, accumulation, and coarse modes. Similar to CESM/CARMA, we run CESM‐MAM from 2016 to 2017 nudged to Modern‐Era Retrospective analysis for Research and Applications meteorology (Rienecker et al., 2011).

In this study we compare the simulations with data on refractory BC measured by a Single‐Particle Soot Photometer during HIPPO from 2009 to 2011 (Schwarz et al., 2010, 2013; Wofsy et al., 2012) and ATom from 2016 to 2017 (Wofsy et al., 2018); sea salt and accumulation mode aerosol mass concentration measured by Particle Analysis by Laser Mass Spectrometry (PALMS; Froyd et al., 2009; Murphy et al., 2006) during ATom; the aerosol size distribution measured by the Aerosol Microphysical Properties instrument package (Kupc et al., 2017; Williamson et al., 2018) during ATom; and size distributions measured by the Printed Optical Particle Spectrometer (Gao et al., 2016) on an instrumented balloon over Kunming, China, in August 2015 (Yu et al., 2017).

## 3 Convective Transport Schemes

In the default convection scheme, strong updrafts within the deep convection (about 5–10 m/s) transport aerosols upward from the cloud base to 9–18 km in one time step (i.e., 30 min). A recent field study (Fan et al., 2018) shows that entrained particles including nucleation‐mode particles (<50 nm in diameter) are activated above the cloud base within the deep convective regions. However, the default convective transport parameterization scheme in CESM does not consider aerosol activation from entrained air above the cloud base (Berg et al., 2015; Wang et al., 2013; Yang et al., 2015). Consequently, the aerosol entrained above the cloud base is efficiently transported upward to the upper troposphere. Note climate models (Koch et al., 2009) that consider hydrophobic dust, BC, and organics might overestimate particles in the upper troposphere since the hydrophobic species are usually not efficiently removed in cloud by convections in these models. For instance, improved BC vertical profiles are simulated by Goddard Earth Observing System‐Chemistry (Wang et al., 2014) when the in‐cloud removal efficiency of hydrophobic BC is largely increased.

In the modified scheme we include aerosol secondary activation above the cloud base following Wang et al. (2013) and Grell and Freitas (2014). The modified scheme is applied to both CARMA and MAM in CESM. The details of the default and modified convective transport schemes in CESM are detailed in Text S1 in the supporting information. Note that CESM/CARMA does not separate hydrophobic and hydrophilic aerosols, and thus, the modified aerosol scheme implicitly removed them both.

## 4 Results

**Figure 1:**
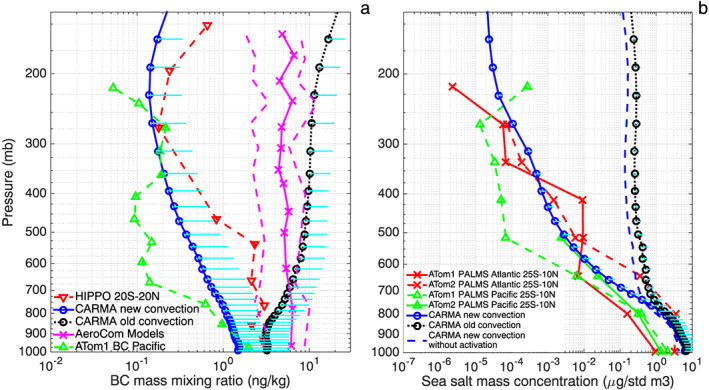
(a) Vertical profiles of black carbon aerosol mass mixing ratio in the tropics (20°S to 20°N) observed during the HIPPO field campaign (red; Schwarz et al., 2010) and ATom1 field campaign (green); simulated tropical profiles over the Pacific Ocean with old (black) and modified (blue) convection schemes in CESM/CARMA. Spatial variabilities (1 standard deviation) from the model are denoted by the cyan lines; simulations by the AeroCom models summarized by Schwarz et al. (2010) are shown in pink (model mean in solid, 25% and 75% in dashed); (b) vertical profiles of sea‐salt aerosol mass concentration (μg std m^−3^) in the tropics (25°S to 10°N) observed by PALMS during ATom field campaigns (red and green; Wofsy et al., 2018); simulated 0.2–3 micron sea‐salt concentration profiles with old (black) and modified (blue solid line) convection schemes by CESM/CARMA. Modeled sea‐salt profile using the modified convection but with activation turned off is denoted by the blue dashed line. Simulations are domain averaged rather than along the flight track, and monthly simulation output when the measurements were made is used. HIPPO = HIAPER Pole‐to‐Pole Observations; CARMA = Community Aerosol and Radiation Model for Atmospheres; AeroCom = Aerosol Comparisons between Observations and Models; ATom = Atmospheric Tomography Mission; PALMS = Particle Analysis by Laser Mass Spectrometry; BC = black carbon; CESM = Community Earth System Model.

Previous studies show that global models overestimate BC mass mixing ratio in the middle and upper troposphere by a factor of 100–1,000 (Koch et al., 2009; Schwarz et al., 2010, 2013). Figure 1a shows HIPPO and ATom1 BC vertical mixing ratio profiles over the tropical Pacific Ocean compared with climatological simulations from CESM/CARMA using the default convective transport scheme (shown by the black dotted line; Yu, Toon, Bardeen, et al., 2015) and with simulations from the Aerosol Comparisons between Observations and Models (AeroCom; Schwarz et al., 2010, 2013; shown by the pink curves). The models simulate a mass mixing ratio of BC that is constant within an order of magnitude from the lower troposphere to 200 hPa, which is inconsistent with the in situ observations measured during HIPPO. Figure 1b illustrates recent PALMS measurements from the NASA ATom field campaign, showing for the first time the vertical profile of sea salt over the tropical ocean from the boundary layer through the upper troposphere. PALMS data show a 4–6 order of magnitude decrease in sea‐salt (0.2–3 μm in diameter) mass mixing ratio from the surface to the upper troposphere, while CESM/CARMA with the default convective scheme shows a mixing ratio of sea salt that is constant above the marine boundary layer within an order of magnitude and overestimates observations by a factor of 1,000 near 200 hPa. The large discrepancy (a factor of 100–1,000) for BC and sea‐salt aerosol between model and observations near 200 hPa suggests that CESM transport aerosol to the upper troposphere too efficiently because they lack sufficient wet removal, especially in tropics where mesoscale deep convection happens frequently all year around.

As shown in Figure 1a, CESM/CARMA with the modified convective scheme reproduces the observed BC mixing ratio above the tropical Pacific Ocean within the variability of the data. In the model, BC is coated with water and other aerosol species (e.g., sea salt, organics, and sulfate) and with the new convective transport scheme it is activated when entrained into the updraft region and is removed from the cloud when precipitation forms. Comparisons of BC vertical distributions between the model and HIPPO observations averaged in other latitude bands are shown in Figure S1. In general, our new scheme reduces model/measurement discrepancies for upper tropospheric BC concentration globally, with the most dramatic improvements in the tropics.

Sea salt is one of the major primary particles emitted near the ocean surface. Figure 1b compares simulations with observations to constrain the convective transport scheme. Salt particles are activated and efficiently removed in cloud with the modified scheme as shown in Figure 1b. We note that the sea‐salt concentration drops by 4–6 orders of magnitude from the surface to 200 hPa, while BC drops by 1–2 orders of magnitude. This difference is because aerosol measurements during HIPPO and ATom were mostly taken over remote oceanic regions where sea salt but not BC is emitted. Therefore, BC is already partially depleted at the surface locations sampled during the aircraft missions. An example of a more polluted region is shown in Figure S2, which plots ATom BC profiles close to central Africa around 20°W. Unlike the marine boundary layer, a marginal difference is found for BC at the surface near the source region between the two different convective transport schemes (Figure S2). As a result of the polluted outflow, the surface concentration near Africa is 2 to 3 orders of magnitude higher than over the Pacific Ocean basin plotted in Figure 1a. The concentrations drop by ~3 orders of magnitude from the surface to the upper troposphere as observed and modeled with the modified convection scheme. Detailed comparisons of simulations using the revised model with ATom sea‐salt vertical distributions averaged over various latitude bands are shown in Figure S3. Only a small difference for sea‐salt vertical profiles is found between the default convection run and the modified convection run but without the secondary activation as shown in Figures 1b and S3. Therefore, the activation from entrained air above the cloud base is essential to reproducing the observed aerosol vertical distribution as also noted in Wang et al. (2013) and Yang et al. (2015).

**Figure 2:**
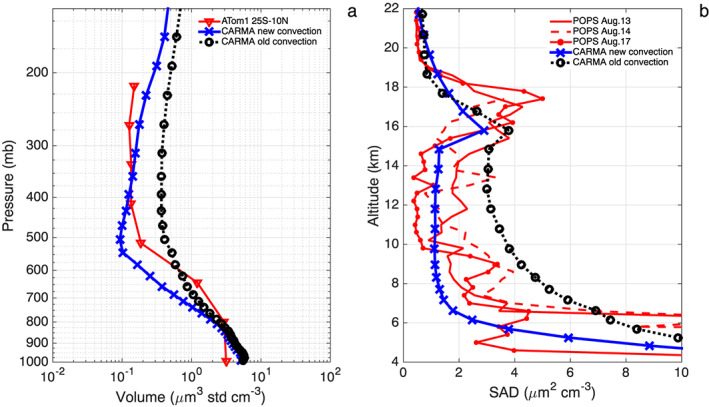
(a) Vertical profiles of the total aerosol volume density (μm^3^/std cm^3^) from 0.06 to 4.5 μm in diameter observed by optical spectrometers (Kupc et al., 2017) during ATom (red); simulated profiles with old (black) and modified (blue) convection schemes in CESM/CARMA; (b) vertical profiles of the total aerosol surface area density (SAD; μm^2^/cm^3^) from 0.14 to 3 μm in diameter observed by POPS (Gao et al., 2016) over Kunming, China in August 2015 (Yu et al., 2017). Simulated SAD profiles with the old and modified convection schemes are shown by black and blue lines, respectively. The simulations are domain averaged rather than along the flight track, and monthly simulation output when the measurements were made is used. ATom = Atmospheric Tomography Mission; CARMA = Community Aerosol and Radiation Model for Atmospheres; POPS = Printed Optical Particle Spectrometer.

Figure 2a shows that the total aerosol volume mixing ratio (for particles from 0.06 to 4.5 μm in diameter) measured over the Pacific and Atlantic Ocean basins in the tropics (25°S to 10°N) during ATom decreases by 1–2 orders of magnitude from the surface to the middle troposphere (500 hPa). The observed volume density then remains constant from 500 hPa to upper troposphere (200 hPa). In contrast, BC and salt mixing ratios (see Figure 1) decrease continuously from the surface to the upper troposphere. The constant aerosol volume density in the middle‐upper troposphere, in contrast to the fast decline of primary aerosol with altitude, suggests in situ formation of secondary aerosol including sulfate and organics. CESM/CARMA predicts similar vertical profiles to those observed with decreasing aerosol volume mixing ratio from the surface to 500 hPa and constant or even increasing mixing ratio with pressures below 500 hPa (Figure 2a). The simulation with the modified convective scheme (in blue) shows a decrease (by a factor up to 5) in aerosol volume mixing ratio density in the middle‐upper troposphere compared with the default model simulation (in black). The modified convective transport simulation provides improved agreement throughout the troposphere.

In a previous study (Yu et al., 2017), we showed that CESM/CARMA overestimated the particle surface area by a factor of 2–3 in the middle and upper troposphere from 6 to 14 km compared with in situ Printed Optical Particle Spectrometer measurements (Gao et al., 2016) over Kunming, China (25°N, 102°E), in August 2015 (Figure 2b). With the modified convective scheme, the aerosol surface area is reduced by a factor of 2–3, yielding better agreement between simulations and observations in the troposphere. The Asian monsoon still transports precursors such as volatile organic compounds and SO_2_ to the upper troposphere, leading to an aerosol enhancement (Asian Tropopause Aerosol Layer) near the tropopause (Vernier et al., 2015; Yu, Toon, Neely, et al., 2015; Yu et al., 2017). The simulated Asian Tropopause Aerosol Layer is in better agreement with the observations using the new convective transport scheme.

Secondary activation from the entrained air removes aerosol effectively, especially in the free troposphere. The global burden of BC decreases by 23% with the modified convection scheme. The calculated partially adjusted effective radiative forcing (ERF; Forster et al., 2016) is +0.39 ± 0.11 W/m^2^ with the original convective transport scheme, which agrees with estimate from Intergovernmental Panel on Climate Change Fifth Assessment Report (Boucher et al., 2013). The ERF with the modified convective scheme drops significantly by 64% to +0.14 ± 0.13 W/m^2^. An earlier study (Samset et al., 2014) adjusted BC vertical profiles from AeroCom Phase II models to observations and found a smaller reduction of 25% on the estimated radiative forcing of BC.

## 5 Discussion

**Figure 3:**
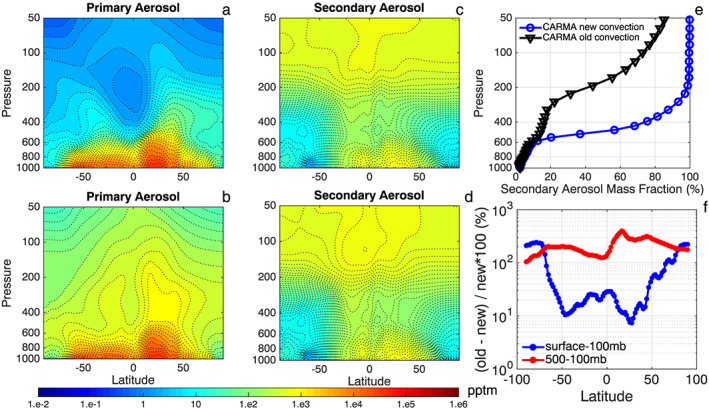
(a) Simulated vertical profiles of zonal averaged primary aerosol mass mixing ratio (the sum of black carbon, dust, salt, and primary organics, unit: pptm) with the modified convection scheme; (b) same as (a) but with the default convection scheme; (c) same as (a) but for secondary particles (sum of sulfate and secondary organics); (d) same as (c) but with the default convection scheme in CESM/CARMA; (e) simulated global mean mass fraction of secondary particles (sulfate and secondary organics aerosols) to the total particles as a function of pressure; (f) fractional change (%) of the column aerosol mass density between the original and the modified convective transport schemes. Simulations averaged in the middle and upper troposphere (above pressure level of 500 hPa) are shown in red; those averaged from the surface to 100 hPa are shown in blue. CARMA = Community Aerosol and Radiation Model for Atmospheres.

Deep convection dominates the vertical transport of aerosol and trace gases emitted or formed near the surface. Using the modified convective transport scheme (see Figure 3a), modeled vertical profiles of the primary aerosol emitted from the surface (sea salt and BC are shown, but dust and primary organics are similarly impacted) show a minimum in the tropical upper troposphere from 300 to 200 hPa with the concentration about a factor of 100–1,000 less than calculated with the default convective transport scheme (see Figure 3b). The impact of the convection scheme on primary aerosol concentrations is significant in middle to high latitudes (a factor of 10 to 100 in the upper troposphere lower than the default scheme) but is not as large as in the tropics due to weaker and less frequent deep convection at those latitudes (Liu & Liu, 2016). We also compared CESM‐CARMA simulations with another aerosol module in CESM (modal aerosol model, MAM; Liu et al., 2012). As shown in Figure S4, the simulations with the default convective transport scheme using either CARMA or MAM aerosol modules show similar magnitudes of aerosol concentration for BC and sea salt in the middle and upper troposphere. The modified convection scheme improves both CARMA and MAM aerosol results (Figure S4). Note that simulations by CARMA and MAM are both domain averaged and thus only simulate the climatology. The default convective transport schemes without the secondary activation disagrees by a few orders of magnitude from the observations. Using the new convective removal scheme, the temporal and spatial mismatch between climatological simulations with numerous field campaigns has uncertainties of much smaller magnitude than with the old scheme (usually within a factor of 2 in the middle and upper troposphere).

As expected, smaller model/measurement differences due to changes in the convection scheme are found for the vertical distributions of secondary particles (e.g., sulfate and secondary organics; see Figures 3c and 3d). Secondary aerosol is formed in the troposphere (in the cold environment above the convection in many cases). Therefore, the vertical distributions of secondary aerosol are less affected by the modified convective removal scheme, although secondary particles are still subject to removal by entrainment.

Since primary particles are removed with the modified convective transport scheme, the mass fraction of the secondary particles increases sharply above the middle troposphere (pressures lower than 600 hPa) and dominates (over 80%) for pressures lower than 400 hPa (Figure 3e). The total aerosol mass simulated in the middle and upper troposphere (100 to 500 hPa) decreases by a factor of 2–3 compared with simulations using the default convection scheme (Figure 3f). The total tropospheric aerosol column burden decreases by 10–20% in midlatitudes and by a factor of 2–3 in polar regions. The polar aerosol burden changes because fewer particles can survive in the convection before being transported cross‐latitude from the source region. Thus, we think the effects on polar aerosol burden from the change in convective transport scheme are indirect. A reduced aerosol column in the case of the modified convective scheme produces less cooling from aerosol scattering, yielding a net top‐of‐atmosphere radiative flux of +1 W/m^2^ compared with the default scheme.

## 6 Summary

Recent field campaign data suggest that the concentrations of global primary aerosols including sea salt and BC drop by 1 to 4 orders of magnitude from the surface to the upper troposphere and lower stratosphere. The default convective scheme in CESM transports aerosol from the surface to the upper troposphere too efficiently and leads to an overestimation of primary aerosol by a factor of 10–1,000 in the global middle and upper troposphere, especially in the tropics. Our study modifies the convective transport scheme following Wang et al. (2013) by considering aerosol activation from entrained air above the cloud base and further removal within clouds when cloud droplets form precipitation. The modified scheme significantly improves model performance when compared with recent observations from aircraft and balloon platforms. Note that we suggest that global models that include the hydrophobic aerosols may overestimate the aerosols in the upper troposphere if those aerosols are not aged fast enough. The total tropospheric aerosol column mass in the middle and upper troposphere appears to be overestimated by a factor of 2–3 with the default convective transport scheme. In situ observations combined with modeling studies suggest that aerosols in the middle and upper troposphere over tropics are mostly secondary. The new measurements and our climate model results suggest that secondary activation above the cloud base in the deep convection can efficiently remove particles. We find that the corrections we applied improve model comparisons with data and likely improve quantification of aerosol‐radiation‐cloud‐climate interactions. While the correction in the convection scheme described here is specific to the CESM model, it describes a potential issue that could impact other models. As was clear in the AeroCom/BC study, there was a mismatch between observations of vertical profiles and a majority of the AeroCom models (Schwarz et al., 2013). These were older models that may have inaccurately transported aerosol in convection; it would be beneficial to examine those comparisons with current state‐of‐the‐art models. Constraining processes in climate models with observations is critical to establish confidence in results for present and future climates. The profile comparisons presented here showed a clear problem with the CESM climate model. The unique feature of the fix presented is that it improved model‐measurement profile agreement for multiple aerosol species and globally, increasing our confidence in its suitability for this problem. We show that the modified convective transport scheme in CESM gives 23% decrease in BC global burden and ~60% decrease in the partially adjusted ERF due to anthropogenic BC. Our study highlights the potential strong overestimation of BC radiative forcing from multiple global models without the robust and realistic convective transport schemes.

## Supporting information



Supporting Information S1
