# Supplementary material for: Efficient In‐Cloud Removal of Aerosols by Deep Convection
Source: Geophys Res Lett. 2019 Jan 23;46(2):1061–9. doi: 10.1029/2018GL080544 (PMC8243348; doi:10.1029/2018GL080544)
Supplement: Supplementary file 1 — Supporting Information S1 [file GRL-46--s001.docx]

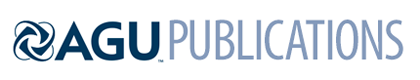


Geophysical Research Letters

Supporting Information for

**Efficient In-cloud Removal of Aerosols by Deep Convection**

Pengfei Yu^1,2,7^, Karl D. Froyd^1,2^, Robert W. Portmann^2^, Owen B. Toon^3,4^, Saulo R. Freitas^8^, Charles G. Bardeen^5^, Charles Brock^2^, Tianyi Fan^6^, Ru-Shan Gao^2^, Joseph M. Katich^1,2^, Agnieszka Kupc^1,2,10^, Shang Liu^9^, Christopher Maloney^3,4^, Daniel M. Murphy^2^, Karen H. Rosenlof^2^, Gregory Schill^1,2^, Joshua P. Schwarz^2^, and Christina Williamson^1,2^

^1^ Cooperative Institute for Research in Environmental Sciences, University of Colorado, Boulder, Colorado, USA.

^2^ Earth System Research Laboratory, National Oceanic and Atmospheric Administration,

Boulder, Colorado, USA.

^3^ Department of Atmospheric and Oceanic Sciences, University of Colorado, Boulder, Colorado, USA.

^4^ Laboratory for Atmospheric and Space Physics, University of Colorado, Boulder, Colorado, USA.

^5^ National Center for Atmospheric Research, Atmospheric Chemistry Division, Boulder, Colorado, USA.

^6^ College of Global Change and Earth System Science, Beijing Normal University, Beijing, China

^7^ Institute for Environment and Climate Research, Jinan University, Guangzhou, China

^8^ Goddard Earth Sciences Technology and Research, Universities Space Research Association, Columbia, MD, USA

^9^ School of Earth and Space Sciences, University of Science and Technology of China, Hefei, Anhui 230026, China

^10^ Now at University of Vienna, Faculty of Physics, Boltzmanngasse 5, 1090 Vienna, Austria

**Contents of this file**

Text S1

Figures S1 to S4

Text S1.

**Convective Transport Schemes**

The default convective parameterization scheme in CESM, a steady-state “plume” model, transports constituents including aerosols and trace gases within the updraft by modeling sub-grid scale entrainment and detrainment as given by Equation 1 [Neale et al., 2010]. However, the in-cloud aerosol wet removal rate is calculated based on the grid-cell mean quantities (denoted in Equation 2).

$-\frac{\partial\left( M_{u}q_{uI} \right)}{\partial p}=E_{u}q_{e}*(1-fwet)-D_{u}q_{uI}$ Eq.1

$\left( \frac{\partial q_{e}}{\partial t} \right)_{wet}=\alpha*fcld*\left( \frac{Prec}{cldm} \right)*q_{e}$ Eq.2

In Eq. 1 and 2, $M_{u}$ denotes air mass velocity within the updraft (hPa/s); $q_{uI}$ denotes tracer mass mixing ratio (for interstitial aerosols, which are those inside a cloud that have not activated into cloud droplets) within the updraft (kg/kg); $q_{e}$ denotes environment or grid-cell mean tracer mass mixing ratio; $fwet$ denotes the fraction of grid-cell mean aerosol being wet removed; $E_{u}$ denotes entrainment rate (1/s) within the updraft; $D_{u}$ denotes detrainment rate (1/s) within the updraft; $p$ denotes atmospheric pressure (hPa);$\left( \frac{\partial q_{e}}{\partial t} \right)_{wet}$denotes the grid cell mean aerosol in-cloud wet deposition rate (kg/kg/s); $fcld$denotes convective cloud area fraction in the grid cell. $Prec$ denotes grid-cell mean precipitation production rate (kg/kg/s); $cldm$ is the grid-cell mean cloud condensate mixing ratio (kg/kg); $\alpha$ denotes the tuning parameter for wet removal of aerosols which is composition dependent (e.g. $\alpha=0.8$ for sea salt; $\alpha=0.2$ for black carbon). The in-cloud removal is performed separately from the steady-state convective transport (Equation 1) before the constituents are redistributed vertically in one time-step (i.e. 30 minutes).

In the modified convective transport scheme shown in Equations 3-8, we treat convective scale aerosol-cloud interaction explicitly (including secondary activation from entrained air above the cloud base) following Wang et al. [2013] and link the modified scheme with the sectional aerosol size bins in and out of the convective clouds [Yu et al., 2015a; Toon et al., 1988]. Note that the convective transport equations in the downdraft are introduced in Neale et al. [2010]. The transport equations for the downdraft are similar to those for updraft except that no aerosol activation is assumed within the downdraft in the modified scheme as air is sub-saturated there [Wang et al., 2013]. These equations differ from the default scheme (Eq.2) in several ways. First, we transport both interstitial (not activated, outside of the cloud droplets) and cloud-borne (activated, within the cloud droplets) aerosols by convection (Eq.3 and Eq.4). Note cloud-borne aerosols are not transported by deep convection in the default treatment. Second, we include an aerosol activation term ($Act*q_{uI}$) in both the interstitial and cloud-borne aerosol convective transport equations (Eq.5). Finally, we include a wet removal term ($Wet*q_{uc}$) in the convective cloud that is a function of precipitation rate ($Prec$) and aerosol mixing ratio in cloud ($cldm$) (Eq.6).

$-\frac{\partial\left( M_{u}q_{uI} \right)}{\partial p}=E_{u}q_{e}-D_{u}q_{uI}-Act*q_{uI}$ Eq.3

$-\frac{\partial\left( M_{u}q_{uc} \right)}{\partial p}=E_{u}q_{ec}-D_{u}q_{uc}+Act*q_{uI}-Wet*q_{uc}$ Eq.4

$Act=ract*M_{u}$ Eq.5

$Wet=\frac{Prec}{cldm}$ Eq.6

$\left( \frac{\partial q_{e}}{\partial t} \right)_{up}=\frac{\partial\left( M_{u}q_{\mathrm{uI}} \right)}{\partial p}- \frac{\partial\left( M_{u}q_{e} \right)}{\partial p}-Act*q_{uI}$ Eq.7

$\left( \frac{\partial q_{\mathrm{ec}}}{\partial t} \right)_{up}=\frac{\partial\left( M_{u}q_{\mathrm{uc}} \right)}{\partial p}-\frac{\partial\left( M_{u}q_{\mathrm{ec}} \right)}{\partial p}+Act*q_{uI}-Wet*q_{uc}$ Eq.8

In these equations, $q_{uc}$ denotes cloud-borne aerosol mixing ratio within the updraft; $q_{ec}$ denotes grid-cell mean cloud-borne aerosol. Note that CESM-CARMA doesn’t track stratiform and convective cloud-borne aerosol separately as in CESM-MAM [Liu et al., 2012; Wang et al., 2013]. We assume that the convection can entrain both convective and stratiform cloud borne aerosol into the sub-grid convective plumes. The activation term, $Act$ (1/s), denotes the rate of aerosol activated to cloud drops within the updrafts; $ract$ denotes a tuning parameter (1/hPa) for activation efficiency, that depends on aerosol type and size. Aerosol activation measured in both clean marine air and polluted continental air by Komppula et al. [2005] suggests that the activation fraction for particles greater than ~0.5 µm in diameter is high (over 90%) and drops quickly with decreasing aerosol size. We set the tuning parameter $ract$ to be 0.04 for any aerosols greater than 0.5 µm in diameter and 0.01 for smaller aerosols. Note that in our present study we assume the secondary activation efficiency does not change with aerosol composition, while in the real atmosphere the secondary activation efficiency may vary. A better quantification of the efficiency is needed in the future using more in-situ and lab experiment data. The updraft flux equation (Eq.3) provides an exponential decay of aerosols due to activation from entrained air above the cloud base, similarly as the in-cloud scavenging efficiency parameterized in previous studies [Rotman et al., 2004; Liu et al., 2001; Wang et al., 2014]; $Wet$ (1/s) denotes wet removal rate of cloud-borne aerosols in the convective scale, which is estimated to first order as the ratio of grid-cell mean precipitation rate and cloud condensate mixing ratio (Eq.6). After the steady-state aerosol mass mixing ratio within the updraft ($q_{uI}$ and $q_{uc}$) are estimated in Eq.3-6, the grid-cell mean aerosol tendencies ($\left( \frac{\partial q_{e}}{\partial t} \right)_{up}$ and $\left( \frac{\partial q_{\mathrm{ec}}}{\partial t} \right)_{up}$, kg/kg/s) due to the updraft portion of the deep convection are calculated by solving the mass conservation equation (Eq.7-8). Note that the grid-cell mean aerosol tendencies due to the downdraft portion of the deep convection remains unchanged from Neale et al. [2010]. The aerosol resuspension due to cloud evaporation within the subgrid convective clouds suggested by Wang et al. [2013] is not considered in current study.

Figure S1 Black carbon mass mixing ratio (ng/kg) vertical profiles averaged in different latitude bands measured by HIPPO in January of 2009 (red for mean, pink for median). Model simulations with the default convective transport scheme are shown in black lines and the modified scheme in blue lines.

Figure S2 Vertical profiles of black carbon aerosol mass mixing ratio (ng/kg) in the tropical Atlantic Ocean (20°S-20°N) observed during the ATom (August 2016) field campaign (red); simulated profiles with old (black) and modified (blue) convection schemes in CESM/CARMA

Figure S3 Sea Salt mass concentration (µg std m^-3^) vertical profiles averaged in different latitude bands measured by PALMS during ATom (August 2016 & Jan-Feb of 2017) (red). Model simulations (submicron particles) with the default convective transport scheme are shown in dotted black lines, the modified scheme in dotted blue lines and the modified scheme without secondary activation in dashed pink line.

Figure S4 Zonal averaged sea salt (left column) and black carbon (right column) concentrations in ambient air (µg m^-3^) with different aerosol modules and convective transport schemes. CESM-CARMA simulations with the modified convective transport scheme are shown in the first row; CESM-CARMA simulations with the default scheme are shown in the second row; CESM-MAM simulations with the modified and default scheme are shown in the third and the fourth row.
